# Small Molecule Inhibitors of the Response Regulator ArsR Exhibit Bactericidal Activity against *Helicobacter pylori*

**DOI:** 10.3390/microorganisms8040503

**Published:** 2020-04-01

**Authors:** Andrés González, Javier Casado, Eduardo Chueca, Sandra Salillas, Adrián Velázquez-Campoy, Javier Sancho, Ángel Lanas

**Affiliations:** 1 Aragon Institute for Health Research (IIS Aragón), San Juan Bosco 13, 50009 Zaragoza, Spain; 2 Institute for Biocomputation and Physics of Complex Systems (BIFI), Mariano Esquilor (Edif. I+D), 50018 Zaragoza, Spain; 3 Department of Biochemistry and Molecular & Cellular Biology, University of Zaragoza, Pedro Cerbuna 12, 50009 Zaragoza, Spain; 4 Centro de Investigación Biomédica en Red de Enfermedades Hepáticas y Digestivas (CIBERehd), Monforte de Lemos 3-5, 28029 Madrid, Spain; 5 Fundación Agencia Aragonesa para la Investigación y el Desarrollo (ARAID), Government of Aragon, Ranillas 1-D, 50018 Zaragoza, Spain; 6 Digestive Diseases Service, University Clinic Hospital Lozano Blesa, San Juan Bosco 15, 50009 Zaragoza, Spain; 7 Department of Medicine, Psychiatry and Dermatology, University of Zaragoza, Pedro Cerbuna 12, 50009 Zaragoza, Spain

**Keywords:** *Helicobacter pylori*, response regulator, ArsR, antimicrobial therapy, U-binders

## Abstract

*Helicobacter pylori* is considered the most prevalent bacterial pathogen in humans. The increasing antibiotic resistance evolved by this microorganism has raised alarm bells worldwide due to the significant reduction in the eradication rates of traditional standard therapies. A major challenge in this antibiotic resistance crisis is the identification of novel microbial targets whose inhibitors can overcome the currently circulating resistome. In the present study, we have validated the use of the essential response regulator ArsR as a novel and promising therapeutic target against *H. pylori* infections. A high-throughput screening of a repurposing chemical library using a fluorescence-based thermal shift assay identified several ArsR binders. At least four of these low-molecular weight compounds noticeably inhibited the DNA binding activity of ArsR and showed bactericidal effects against antibiotic-resistant strains of *H. pylori*. Among the ArsR inhibitors, a human secondary bile acid, lithocholic acid, quickly destroyed *H. pylori* cells and exhibited partial synergistic action in combination with clarithromycin or levofloxacin, while the antimicrobial effect of this compound against representative members of the normal human microbiota such as *Escherichia coli* and *Staphylococcus epidermidis* appeared irrelevant. Our results enhance the battery of novel therapeutic tools against refractory infections caused by multidrug-resistant *H. pylori* strains.

## 1. Introduction

Multidrug resistance in most clinically relevant bacterial pathogens is a major global public health concern [1,2]. Focusing on new molecular targets could lead to the development of novel classes of antibacterial drugs that avoid the resistance strategies evolved by microorganisms to existing antibiotics. In vivo essential genes represent a powerful source of promising targets for antibacterial drug development [3]. Among the plethora of genes indispensable for the growth and survival of bacterial pathogens in the host, transcriptional regulators (TRs) stand out because of the potential multitargeting effect of anti-TR molecules on cellular virulence and physiology. Anti-TR drugs will inhibit not only the activity of TRs, but will also potentially affect the expression of the downstream genes into the regulatory network. Hence, anti-TRs drugs could function both as bactericidal/bacteriostatic chemotherapy but also as antivirulence strategies, avoiding the expression of toxins, adhesins, invasins, capsule, quorum-sensing mechanisms and efflux pumps and thereby disarming the pathogen, and enhancing its susceptibility to the host immune response or to the action of conventional antibiotics [4].

The World Health Organization has included *Helicobacter pylori* on its “high-priority” list of antibiotic-resistant bacteria that pose the greatest threat to human health and for which there is an urgent need for new antibiotics [5]. In *H. pylori*, the OmpR-like transcriptional regulator ArsR is part of the acidic-responsive ArsRS two-component system (also known as HP165-HP166 system), comprised the sensor histidine kinase ArsS (HP165) and the response regulator ArsR (HP166) [6]. By this signal transduction mechanism, the acidification of the *H. pylori* periplasm into the human stomach is sensed by ArsS via protonation of histidine residues of the periplasmic sensory domain [7]. This environmental signal triggers auto-phosphorylation of ArsS and trans-phosphorylation of the response regulator ArsR, which consequently increases its DNA-binding affinity toward a set of acid-responsive target promoters [8]. The ArsRS two-component system is critical for both acid acclimation and stomach colonization of *H. pylori*. The expression of several key players involved in the acid resistance such as the urease system, the inner membrane channel protein UreI, the arginase RocF, the alpha-carbonic anhydrase HP1186 as well as the aliphatic amidases AmiE and AmiF appears transcriptionally regulated by the phosphorylated form of ArsR (ArsR∼P) [6,9,10]. Additionally, the ArsRS two-component system controls the expression of major virulence factors of *H. pylori* including adhesins and biofilm [11,12,13,14]. Notably, while the histidine kinase ArsS null mutants are viable in vitro, the response regulator ArsR appears essential for *H. pylori* viability, suggesting that a subset of essential genes included in the ArsR regulon can be regulated by a non-phosphorylated form of ArsR [8,15]. Thus, as an essential protein for microbial viability, easily produced under lab conditions and with no counterpart in humans, the response regulator ArsR constitutes a promising therapeutic target against *H. pylori* infection. 

In previous studies, we have validated the use of the *H. pylori* orphan response regulator HsrA as an effective therapeutic target for the development of novel bactericidal antimicrobials against this clinically relevant pathogen [16,17]. In the present study, we screened the Prestwick Chemical Library^®^, a collection of 1120 FDA-approved, off-patent, small-molecule drugs for compounds that specifically bind to ArsR and potentially inhibit its essential function. At least four non-antibiotic drugs noticeably inhibited the DNA binding activity of ArsR and exhibited bactericidal activities against *H. pylori*. 

## 2. Materials and Methods 

### 2.1. Bacterial Strains, Culture Media and Growth Conditions

*H. pylori* reference strains ATCC 700392, ATCC 43504 (metronidazole-resistant), and ATCC 700684 (clarithromycin resistant) were purchased from the American Type Culture Collection (Rockville, MD, USA). The strains were routinely grown in Blood Agar Base No. 2 (OXOID, Basingstoke, UK) supplemented with 8% defibrinated horse blood (OXOID) in a humidified microaerobic incubator (85% N_2_, 10% CO*_2_*, 5% O_2_) at 37 °C for 48–72 h. For certain experiments, bacteria were grown for 48–72 h at 37 °C in brain heart infusion broth (OXOID) supplemented with 4% foetal bovine serum (Gibco, Carlsbad, CA, USA).

*Escherichia coli* strain ATCC 25922 and *Staphylococcus epidermidis* strain ATCC 12228, obtained from the local culture collection of the Department of Microbiology, Preventive Medicine and Public Health of the University of Zaragoza (Spain), were used in some susceptibility evaluations. For this purpose, the strains were grown in Mueller-Hinton agar/broth (PanReac AppliChem, Barcelona, Spain) overnight at 37 °C. 

### 2.2. Chemicals

The Prestwick Chemical Library^®^ was purchased from Prestwick Chemical (Illkirch, France). Aliquots of the 1120 small molecule drugs were provided as 10 mM solutions in 100% dimethyl sulfoxide (DMSO) distributed in 96-well plates, which were stored at −20 °C until use. For some assays, compounds of interest were purchased from Sigma-Aldrich (Saint Louis, MO, USA) and properly stored according to the manufacturer indications. Stock solutions of each drug were freshly prepared at 20 mM in 100% DMSO for electrophoretic mobility shift assays and isothermal titration calorimetry analyses, and at 10.24 g/L in 100% DMSO for minimal inhibitory concentration (MIC)/minimal bactericidal concentration (MBC) determinations. Metronidazole, clarithromycin and ampicillin were purchased from Sigma-Aldrich. Stock solutions of these antibiotics in 100% DMSO were prepared at 10.24 g/L and stored at −20 °C for up to 30 days.

### 2.3. Recombinant Expression and Purification of the H. pylori Response Regulator ArsR

The complete sequence of gene *arsR* was amplified from *H. pylori* strain 26695 (ATCC 700392), cloned into the vector pET-28a (EMD Biosciences, San Diego, CA, USA) and overexpressed in *Escherichia coli* BL21(DE3) (EMD Biosciences). His-tagged ArsR was purified by immobilized metal-affinity chromatography (IMAC) using Zn^2+^ charged Chelating Sepharose Fast Flow resin (GE Healthcare, Chicago, IL, USA) according to the standard protocols. Cell pellets were sonicated in binding buffer (50 mM Tris-HCl (pH 8), 500 mM NaCl, 10% glycerol, 1 mM dithiothreitol (DTT)) supplemented with 1 mM phenylmethylsulfonyl fluoride (PMSF) and 10 mM imidazole. His-tagged ArsR was eluted in the above binding buffer using an imidazole gradient and finally dialyzed in 50 mM Tris-HCl (pH 8), 300 mM NaCl, 10% glycerol, 1 mM DTT. The His-tag was removed by thrombin digestion and the cleaved ArsR protein was conserved at −20 °C in 50 mM Tris-HCl (pH 8), 300 mM NaCl, 10% glycerol. Protein concentration was determined using the BCA™ Protein Assay kit (Thermo Fisher Scientific, Bothell, WA, USA).

### 2.4. High-Throughput Screening for ArsR Binders

A high-throughput screening (HTS) of the Prestwick repurposing collection for molecules that specifically bind to the *H. pylori* ArsR regulator was assessed as previously described by using a fluorescence-based thermal shift assay [18,19]. Previous to the HTS, the biological activity of recombinant ArsR was verified by electrophoretic mobility shift assay (EMSA) as described below. For the HTS, a reaction mixture containing 10 µM of recombinant ArsR protein in buffer 50 mM Tris-HCl (pH 8), 150 mM NaCl, 10% glycerol, 5 mM DTT, and SYPRO^®^ Orange ready-to-use fluorescent stain (Thermo Fisher Scientific) at a final concentration of 10× was freshly prepared. By using V-shape 96-well plates, the mixture of protein and fluorescence stain was put in contact with 250 μM (final concentration) of each compound from the chemical library, while several wells in the microtiter plate were used as reference controls. In these control wells, solution of protein and fluorescence stain was mixed with DMSO (vehicle) instead of compounds. Unfolding curves corresponding to all the wells of the plate were registered from 25 °C to 75 °C in 1 °C steps using a FluoDia T70 High Temperature Fluorescence Microplate Reader (Photon Technology International, Birmingham, NJ, USA), and then analysed using a homemade software that calculates the experimental midpoint temperature of unfolding (*T*_m_) of each well. Compounds that changed thermal stability of the protein above the two-fold standard deviation value of reference controls were identified as ArsR binders. The *T*_m_ values of ArsR in absence and presence of its binders were more accurately calculated using equations for a two-state thermal unfolding model [20].

### 2.5. Electrophoretic Mobility Shift Assays

EMSA experiments were performed as previously described [16] with slight modifications. Briefly, a 300-bp promoter region of the *H. pylori rocF* (arginase) gene was amplified by PCR and used as target DNA sequence of ArsR [6] in all EMSA experiments. In vitro phosphorylation of recombinant ArsR was performed by incubating the protein during 30 min at 25 °C in phosphorylation buffer containing 50 mM Tris-HCl (pH 7.5), 5 mM MgCl_2_, 50 mM KCl, 1 mM DTT in the presence of 50 mM acetyl phosphate [21]. Prior to the inhibition studies, the in vitro DNA binding activity of the protein was titrated in order to determine the minimum concentration of the protein needed to completely bound 120 ng of the target DNA in a 20 μL reaction volume containing 10 mM Tris-HCl (pH 7.5), 50 mM KCl, 1mM DTT, 5 mM MgCl_2_, 2.5% glycerol. Next, inhibition studies were carried out by mixing proper amounts of ArsR~P and target DNA in the presence of 1, 0.1 and 0.01 mM of each ArsR binder that resulted from the HTS. Binding assays using DMSO instead of compounds were included as vehicle controls. Non-specific competitor DNA corresponding to a 150-bp fragment of gene *pkn22* (*alr2502*) from the cyanobacterium *Anabaena* sp. PCC 7120 was included in all assays. 

Inhibition studies of the HsrA activity by selected ArsR binders were performed as previously described [16,17]. Briefly, 120 ng of *H. pylori porGDAB* promoter were mixed with 6 μM of recombinant HsrA in the presence of 1 mM of each ArsR binders. Mixtures of DNA and protein in a 20 μL reaction volume containing 10 mM bis-Tris (pH 7.5), 40 mM KCl, 100 mg/L BSA, 1 mM DTT and 5% glycerol were incubated at room temperature for 20 min. *pkn22* (*alr2502*) from *Anabaena* sp. PCC 7120 was included non-specific competitor DNA. 

All EMSA analyses were performed using 6% native polyacrylamide gel electrophoresis. Gels were stained with SYBR Safe^®^ (Thermo Fisher Scientific) and analysed by using the Gel Doc 2000 imaging system (Bio-Rad Laboratories, Hercules, CA, USA).

### 2.6. Isothermal Titration Calorimetry Assays

Isothermal titration calorimetry (ITC) studies of the interactions between ArsR and its small molecule inhibitors were carried out as previously described [16]. Solutions of 20 µM of recombinant ArsR and 200 µM of each ligand were freshly prepared using the same buffer containing 50 mM Tris-HCl (pH 8), 150 mM NaCl, 10% glycerol and 1% DMSO. The solution of ArsR in the calorimetric cell was titrated at 25 °C with the solution of each ligand located in the injecting syringe of an Auto-iTC200 calorimeter (MicroCal, Malvern Instruments, Malvern, Worcestershire, UK). A sequence of 19 injections of 2 µL volume was programmed with a time spacing of 150 s, a stirring speed of 750 rpm, and a reference power of 10 µcal/s in the sample cell [22]. Thermodynamic parameters were calculated by non-linear least squares regression data analysis considering a single ligand binding site implemented in Origin 7.0 software (OriginLab, Northampton, MA, USA).

### 2.7. Minimal Inhibitory and Bactericidal Concentrations

Minimal inhibitory concentration (MIC) of the ArsR inhibitors against three different strains of *H. pylori* were determined by the microdilution method as previously described [16], with slight modifications. Briefly, the *H. pylori* reference strains ATCC 700392 (26695), ATCC 43504 (metronidazole-resistant), and ATCC 700684 (clarithromycin resistant) were grown on Blood Agar Base Nº2 (OXOID) supplemented with 8% defibrinated horse blood (OXOID) at 37 °C during 48 h under microaerobic conditions (85% N_2_, 10% CO_2_, 5% O_2_). Bacterial inoculums were freshly prepared by resuspending the agar growth in brain heart infusion (BHI) broth supplemented with 4% foetal bovine serum (FBS) and next diluted to OD_600_ = 0.01 (~10^6^ CFU/mL) in the same medium. ArsR inhibitors purchased from Sigma-Aldrich (Saint Louis, MO, USA) were dissolved at 10.24 g/L in 100% DMSO and then two-fold serially diluted in sterile 96-well flat-bottom microtiter plates using the above freshly prepared bacterial inoculum. Thus, a range of concentrations from 256 to 0.125 mg/L was tested for each ArsR inhibitor against each *H. pylori* strain. DMSO (vehicle), metronidazole and clarithromycin were included as controls in all assays. Plates were incubated under microaerobic conditions at 37 °C for 72 h. MIC values were defined as the lowest concentration of compound that completely inhibited the visible growth of bacteria. MBC values, defined as the lowest concentration of compound that prevented the growth of ≥99.9% of *H. pylori* cells, were determined by seeding 10 μL aliquots of each of the two dilutions around the MIC values on Blood Agar Base No. 2 supplemented with 8% of defibrinated horse blood. Plates were incubated during 72 h at 37 °C under microaerobic conditions. Each experiment was performed twice in triplicate. 

MIC and MBC values of selected compounds were also determined against *Escherichia coli* strain ATCC 25922 and *Staphylococcus epidermidis* strain ATCC 12228, according to the EUCAST Guidelines [23]. Bacteria were grown on Mueller-Hinton agar (PanReac AppliChem) overnight at 37 °C. Several typical colonies were then suspended in sterile saline and adjusted to the 0.5 McFarland standard turbidity (~1.5 × 10^8^ CFU per mL). Final inoculum suspensions at 5 × 10^5^ CFU per mL in Mueller-Hinton broth were faced to increasing concentrations of selected ArsR inhibitors in a range of 256 to 0.125 mg/L by the method of microdilution using sterile 96-well flat-bottom microtiter plates. DMSO and ampicillin were included as controls in all assays. Plates were incubated at 37 °C overnight and then MIC values were visually defined by turbidity. For MBC determinations, 10 μL aliquots of each of the two dilutions around the MIC values were seeded on Mueller-Hinton agar and plates were incubated at 37 °C overnight. Experiments were performed twice in triplicate.

### 2.8. Time-Kill Kinetics Assays

Time-kill kinetics assays were carried out as previously described [16]. A bacterial suspension of ~10^6^ CFU/mL of *H. pylori* strain ATCC 700684 was freshly prepared in BHI+FBS from a 48 h culture in Blood Agar Base No. 2 (OXOID) supplemented with 8% defibrinated horse blood (OXOID). 200 µL of this inoculum were faced in triplicate with two concentrations (2-fold MIC and 4-fold MIC) of selected ArsR inhibitors. Plates were incubated under microaerobic conditions (85% N_2_, 10% CO_2_, 5% O_2_) at 37 °C with shaking. Mixtures of bacteria with the same amount of DMSO (vehicle) instead of an ArsR inhibitor were incubated under the same conditions and included as controls in all determinations. Colony forming units (CFUs) were determined at 0, 2, 4, 8, 24 and 32 h after drug exposition by seeding on inhibitor-free blood agar. Experiments were repeated at least twice and the results were presented as log_10_ CFU/mL versus incubation time. Statistical significances were considered if *p* < 0.05 according to the Mann–Whitney *U* test.

### 2.9. Checkerboard Assays

The effect of combinatory action of selected ArsR inhibitors with metronidazole and clarithromycin on the *H. pylori* growth were tested by using the checkerboard assay [16]. 2-fold dilutions of clarithromycin or metronidazole in BHI+FBS were mixed with 2-fold dilutions of each selected inhibitor in 96-well flat-bottom microtiter plates. To prepare the checkerboard plate, serial dilutions of both antimicrobials were firstly prepared using two different sterile microtiter plates, one compound was diluted along the rows in a first plate, and the other compound was diluted along the columns of a second plate. Then, both gradients were mixed in a third microtiter plate and inoculated with a freshly prepared bacterial suspension of *H. pylori* at 2 × 10^6^ CFU/mL in BHI+FBS. After 48 h of incubation at 37 °C under microaerobic conditions, microbial growth was colorimetric, revealed by the addition of 0.1 mg/mL resazurin (Sigma-Aldrich). The interaction between drugs was determined according to the fractional inhibitory concentration index (FICI): FIC_A_ (MIC_A_ in the presence of B/MIC_A_ alone) + FIC_B_ (MIC_B_ in the presence of A/MIC_B_ alone) [24].

## 3. Results

### 3.1. Screening of a Repurposing Chemical Library Identified Several ArsR Binders

During our HTS screening of the Prestwick chemical library based on the thermal shift assay (ligand-induced stabilization/destabilization against thermal denaturation) [19,25], we defined as relevant compounds those that bind to the protein and alter its thermal stability above the twofold standard deviation of *T*_m_ values of reference controls (protein plus DMSO). This definition for ArsR binders included both stabilizing and destabilizing compounds. Following this selection criterion, the target-based HTS approach identified 10 small molecules within the 1120 compound collection (0.9%) as ArsR binders (Table 1).

**Figure 1:**
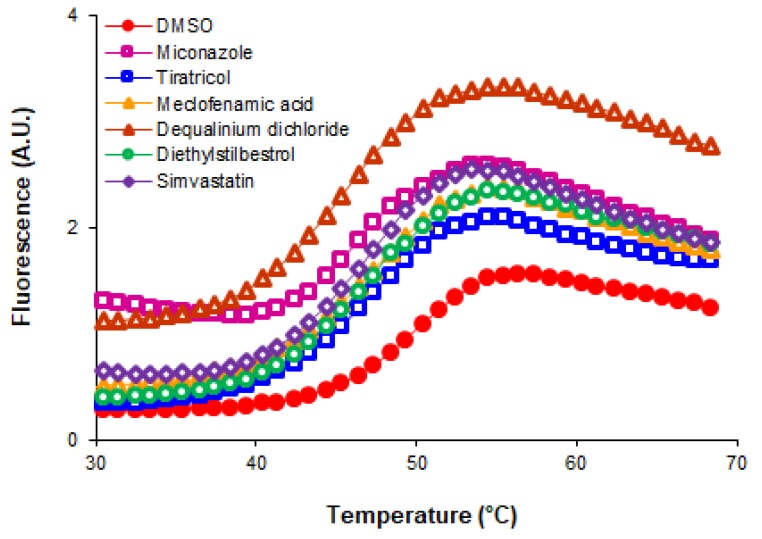
Temperature denaturation profiles of ArsR without added ligands (DMSO) and after the addition of 250 µM of the several ligands (open symbols). Data points (symbols) are experimental observations; the lines are fits to a two-state unfolding model.

Notably, all the ArsR binders identified in our HTS acted as destabilizers of ArsR. In all cases, the presence of the small molecule ArsR binder led to a decrease in the *T*_m_ values of the complexes compared to the *T*_m_ of the protein alone (only with vehicle, DMSO). The decreases in the *T*_m_ values observed with each ArsR binder are indicated in Table 1. The experimental temperature denaturation curves of the ArsR protein with various added ligands are showed in Figure 1. The midpoint of the transition without any ligand (49 °C) is equal to the *T*_m_ of the protein. The addition of 250 µM of ligands shifted the *T*_m_ downward in all cases.

### 3.2. Some Non-Antibiotic FDA-Approved Drugs Inhibited the DNA Binding Activity of ArsR

**Figure 2:**
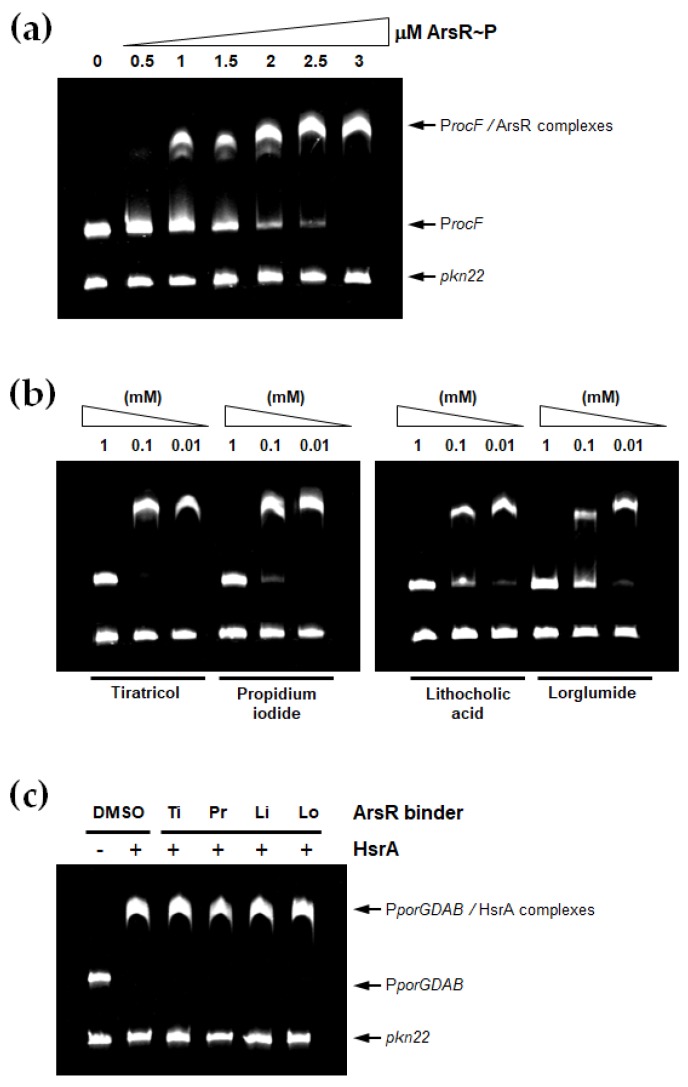
Non-antibiotic FDA-approved drugs inhibited the DNA binding activity of ArsR. (**a**) EMSAs showing the ability of the in vitro phosphorylated regulator (ArsR~P) to specifically bind the promoter region of target *rocF* gene. Increasing concentrations of ArsR~P (indicated in μM) were mixed with 120 ng of target promoter and separated on a 6% PAGE. The *Anabaena* gene *pkn22* was included as non-specific competitor DNA in all assays. (**b**) DNA fragments were mixed with 3 μM of recombinant ArsR~P in the presence of 1, 0.1 and 0.01 mM of each ArsR binder. (**c**) 120 ng of the promoter region of *porGDAB* operon were mixed with 6 μM of recombinant HsrA protein in the absence (DMSO) or presence of 1 mM of tiratricol (Ti), propidium iodide (Pr), lithocholic acid (Li) or lorglumide (Lo), and separated on a 6% PAGE. The *Anabaena* gene *pkn22* was included as non-specific competitor DNA in all assays.

EMSA analyses were carried out in order to define which ArsR binder exhibit the ability of inhibiting the in vitro DNA binding activity of this *H. pylori* essential transcriptional regulator. Previous titration of the protein activity showed that 3 µM of the in vitro phosphorylated regulator (ArsR~P) were sufficient to completely and specifically bound 120 ng of the target DNA promoter under the experimental conditions used in the EMSA (Figure 2a). Consequently, this protein concentration was subsequently used for EMSA inhibition assays by mixing the transcriptional regulator with its target DNA in the presence of 0.01 to 1 mM of each potential inhibitor.

As shown in Figure 2b, only four small molecule compounds from the Prestwick library noticeably inhibited the DNA binding activity of ArsR. Thus, tiratricol, propidium iodide, lithocholic acid and lorglumide completely inhibited the activity of 3 µM of protein at 1 mM of inhibitor. Lithocholic acid and lorglumide affected the activity of the protein even at 10 µM of each compound according to EMSA. No appreciable inhibition of the transcriptional regulator activity was observed in the presence of 1 mM of miconazole, meclofenamic acid, simvastatin, dequalinium dichloride, diethylstilbestrol or dienestrol under the same experimental conditions (data not shown). 

To further demonstrate the specific interactions of the ArsR inhibitors with their target protein, we analysed the inhibition capabilities of these compounds on the biological activity of other *H. pylori* transcriptional regulator, the essential protein HsrA. As shown in Figure 2c, none of the ArsR inhibitors identified here diminished the DNA binding affinity of HsrA by its target promoter. Hence, the interaction and inhibitory effect of DNA binding activity of these small-molecule drugs appeared specific for the response regulator ArsR. 

### 3.3. ArsR Inhibitors Bound to the Protein at 1:1 Stoichiometry in the Micromolar Range

Thermodynamic parameters of the molecular interaction between ArsR and its inhibitors, including reaction stoichiometry (n), dissociation constant (Kd), enthalpy (ΔH) and Gibbs free energy of ligand binding (ΔG) were determined by ITC measurements. As shown in Table 2 and Figure S1 (Supplementary Materials), all the ArsR inhibitors bound to the protein following a 1:1 stoichiometry. Hence, each ArsR monomer appeared to bind one molecule of inhibitor. Despite ITC data which indicated dissociation constants in the micromolar range in all cases, small differences were observed in the binding affinities of the four inhibitors to the target protein (Table 2).

### 3.4. ArsR Inhibitors Exhibited Bactericidal Activities against H. pylori

Whether the inhibition of ArsR activity registered by EMSA has an antimicrobial effect on *H. pylori* viability was determined by means of MIC and MBC calculations. Three different strains of *H. pylori*, including metronidazole- and clarithromycin-resistant strains, were exposed to a range of concentration between 256 to 0.125 mg/L of each ArsR inhibitor by the method of broth microdilution. In all assays, the solvent of the inhibitors (DMSO) as well as the conventional antibiotics metronidazole and clarithromycin were included as controls. As shown in Table 3, all the ArsR inhibitors appeared bactericidal at concentration ≤ 64 mg/L, with MIC values in the range of 16 to 64 mg/L. Propidium iodide and lithocholic acid were slightly more effective than tiratricol and lorglumide as anti-*H. pylori* drugs. In fact, despite the bactericidal effects exhibited by all the ArsR inhibitors appearing at concentrations quite a lot higher than the EUCAST MIC breakpoints of the antimicrobials traditionally used in anti-*H. pylori* therapies [26], it was interesting to note that both propidium iodide and lithocholic acid were more effective than metronidazole and clarithromycin against their respective resistant strains. This fact potentially includes these drugs in the battery of alternative antimicrobial tools for novel personalized therapies against refractory infections caused by antibiotic-resistant strains of *H. pylori*. However, according to their quite low bactericidal activities, we considered tiratricol and lorglumide scarcely recommended as novel potential anti-*H. pylori* drugs.

**Figure 3:**
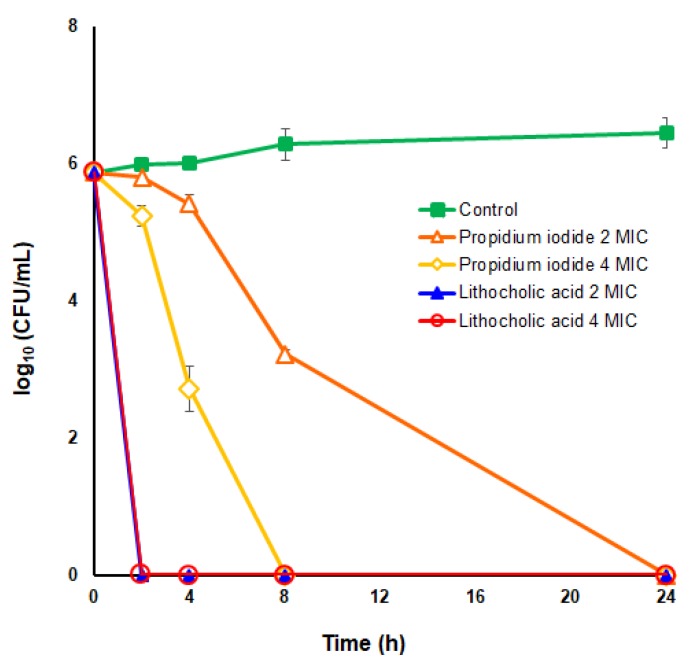
Time-kill kinetics of propidium iodide and lithocholic acid against *H. pylori* strain ATCC 700684. Bacterial counts were determined at time zero and after 2, 4, 8, and 24 hours of incubation with 2× MIC and 4× MIC of each drug. Mixtures of bacteria with DMSO (vehicle) instead of the drug were used as controls. Values are the averages of three independent determinations; vertical bars represent standard deviations. Please note that in some instances the error is smaller than the symbols used.

To further analyze the anti-*H. pylori* bactericidal potential of propidium iodide and lithocholic acid, time-kill kinetic analyses were carried out for these two small ArsR inhibitors against the *H. pylori* strain ATCC 700684 at two different concentrations of each drug, 2× and 4× their MIC values. Notably, bacterial death was significantly faster in the presence of lithocholic acid with independence of the concentration used above the MIC value of the drug. As shown in Figure 3, lithocholic acid was completely lethal for *H. pylori* after only 2 h of exposition with either 2-fold or 4-fold the MIC value. By contrast, the kinetic of microbial death caused by propidium iodide appeared proportional to the concentration of the drug, although the bactericidal effect observed with 4-fold the MIC value was significantly higher (*p* < 0.05) than that observed at 2-fold MIC only after 4 h of drug exposition. Both drugs completely killed the bacterial suspension after 24 h of contact with the microorganism. Since propidium iodide is a well-recognized DNA intercalating agent [27], its use as an antimicrobial drug is quite improbable. Hence, we focused our further analyses to discern the potentiality of lithocholic acid as a novel anti-*H. pylori* drug.

### 3.5. Lithocholic Acid Exhibits Low Antimicrobial Activities against Members of Human Normal Microbiota

Lithocholic acid exhibited a fast bactericidal effect against *H. pylori*, with MBC values of 32 mg/L even on antibiotic (clarithromycin or metronidazole) resistant strains. After only 2 hours of exposition to 2-fold MIC of this drug, a bacterial suspension of ~10^6^ CFU/mL of the *H. pylori* clarithromycin resistant strain ATCC 700684 was completely destroyed. Since lithocholic acid is a secondary bile acid which could cause unspecific damage to bacterial cytoplasmic membranes due to its detergent capacity, we analyse the antimicrobial activity of this drug against two different microorganisms representative of the normal human microbiota, the Gram-negative facultative anaerobe *E. coli*, and the Gram-positive facultative anaerobe *S. epidermidis*. ATCC reference strains of both microorganisms were faced to increasing concentrations of lithocholic acid in a range of 256 to 0.125 mg/L and MIC values were determined by the method of microdilution according to the EUCAST Guidelines [23]. Lithocholic acid exhibited very low antibacterial activities against these two commensal species, with MBC > 256 mg/L against *E. coli* ATCC 25922 and MBC = 125 mg/L against *S. epidermidis* ATCC 12228. Thus, the higher bactericidal effect exerted by this drug against *H. pylori* strains appears to be the result of a specific mechanism of action (e.g., inhibition of the essential regulator ArsR) rather than unspecific antibacterial action such as membrane damage.

### 3.6. Lithocholic Acid Partially Synergizes with Other Antimicrobial Drugs against H. pylori

We analysed the in vitro bactericidal effect of lithocholic acid in combination with other recognized anti-*H. pylori* substances including the conventional antibiotics clarithromycin, metronidazole and levofloxacin, as well as the flavonoid chrysin [16], by the checkerboard method [16,17,24,28]. According to this approach, when two antimicrobial substances are used together in a fixed concentration ratio against a certain microbial strain, and the combinatory action of both drugs noticeably increases the activity of each drug when they act separately against the same strain, we could infer that these substances exhibit a synergistic interaction in their antimicrobial action. In practice, the level of interaction is estimated by calculating how a drug is able to diminish the MIC value of the other drug and vice versa, resulting in a numeric value known as fractional inhibitory concentration index (FICI). To express synergistic interaction (FICI ≤ 0.5), each drug must diminish the MIC value of the other drug in at least two 2-fold dilutions (FIC_drug A_ = 0.25, where FIC_drugA_ = MIC_A in the presence of B_/MIC_A alone_). Notably, in certain drug combinations, one compound noticeably diminishes the MIC of the other drug, but the contrary effect is not observed. In those cases, we could observe a partial synergy known as an “additive effect”, which occurs when the FICI value is >0.5 but ≤1. Hence, the term “synergy” implies that the resulting effect of a combination is significantly greater than the sum of its individual parts, while “additive effect” occurs when substances added together will improve or increase their efficacies, albeit not to the extent of a synergistic interaction [24,28]. As shown in Table 4, lithocholic acid showed additive (partial synergy) interactions with clarithromycin, levofloxacin and chrysin, but it did not interact with metronidazole. Notably, the addition of lithocholic acid reduced the MIC value of clarithromycin up to 32 times (FIC = 0.031). Thus, lithocholic acid noticeably improved the antimicrobial activities against *H. pylori* of the conventional antibiotic clarithromycin and the natural flavonoid chrysin, but the contrary effect was only modest.

## 4. Discussion

With the increasing emergence of multidrug-resistant, extensively drug-resistant and even pandrug-resistant isolates of clinically relevant pathogens worldwide [29,30], drug repurposing provides an alternative method for rapid identification and development of new antibacterial agents, reducing significantly the time and cost of the overall drug development process. The goal of the present study was to identify novel bactericidal activities against *H. pylori* from a collection of drugs that have been approved for other therapeutic uses, taking the essential response regulator ArsR as a molecular target. For the search of drug repurposing candidates, we screened the Prestwick Chemical Library^®^, which consists of 212 FDA-approved off-patent antimicrobials, antiseptics, and antifungals and 1068 miscellaneous low-molecular-weight drugs with a wide range of functions and mechanisms of action and well-characterized pharmacological and toxicological properties. The HTS of the Prestwick library was carried out using a fluorescence-based thermal shift assay [18,19], which operates on the principle that ligand binding alters the thermal stability of proteins. With this method, any compound of the chemical library that preferentially binds to the native state of ArsR increases the protein conformational stability and causes a shift of the protein unfolding curve to higher temperatures due to the increased melting temperature (*T*_m_) of the protein-binder complex. However, some ligands (known as U-binders) destabilize proteins by binding primarily or more strongly to their unfolded states [25], causing a shift of the protein unfolding curves to lower temperatures due to a decrement in the *T*_m_ value of the complexes. Our study identified 10 small molecules within the 1120 compound collection (0.9%) as ArsR U-binders, which decreased the thermal stability of ArsR in a range of 1.6 to 6.8 °C.

Most drug candidates that act as protein ligands increase the thermal stability of targets upon binding [16,18,31,32]. These low molecular weight ligands (known as N-binders) inhibited the activity of essential proteins by binding to and destabilizing or blocking the active site of enzymes, redox proteins, membrane transporters or transcription factors. In the case of bacterial transcriptional regulators, N-binders could inhibit or alter protein dimerization, prevent protein phosphorylation or directly block the binding to target DNA promoters by steric impediment or interaction with amino acid residues involved in the DNA binding motif structure [4,16,17,33,34,35]. Thus, several N-binders of the *H. pylori* essential response regulator HsrA have previously demonstrated potent bactericidal activities against different antibiotic-resistant strains of this pathogen. Such drug candidates appeared to bind the response regulator by its C-terminal effector domain, thereby blocking its essential DNA binding activity [16,17]. 

The antimicrobial activity of the ArsR U-binders identified here occurs by other mechanisms. Under physiological conditions, both in in vitro and in vivo environments, proteins exist in equilibrium between populations of folded (native) and unfolded states. In the case where a particular ligand binds primarily or more strongly to the unfolded state of the protein, upon addition of the ligand the law of mass action dictates that as the ligand becomes bound, the equilibrium will be restored by unfolding molecules from the folded population; hence, these types of ligands (U-ligands) destabilize proteins. Since protein function depends on attaining the native conformation, U-ligands will decrease protein activity by reducing the population of folded proteins. Noticeable reduction in the native population of essential proteins like ArsR lead to lethal disorders in the pathogen physiology. Hence, U-binders have been recognized as promising drug candidates for the treatment of viral and bacterial infections [36,37,38,39], but also cancer [40,41].

Taking into account the EUCAST MIC breakpoints of the antimicrobials used in current anti-*H. pylori* therapies [26], some of the U-binders of ArsR identified here such as propidium iodide and lithocholic acid demonstrated moderate antimicrobial activities against *H. pylori* according to their MIC/MBC values (in the range of 16 to 32 mg/L). However, these two ArsR U-binders appeared more effective than metronidazole and clarithromycin against their respective resistant strains. In addition, the secondary bile acid, lithocholic acid (LCA), exhibited a fast bactericidal effect against *H. pylori*, killing ~10^6^ CFU/mL of the *H. pylori* clarithromycin resistant strain ATCC 700684 after only two hours of exposition to 2-fold MIC (64 mg/L) of this drug. At this concentration, the bactericidal effect of LCA against both Gram-positive and Gram-negative representative microorganisms of the human microbiota appeared irrelevant, supporting a specific mechanism of action against *H. pylori* and guaranteeing minor side effects as a potential novel antimicrobial drug. LCA is formed by the 7-α-dehydroxylation of the primary bile acid, chenodeoxycholic acid (CDCA), as a result of the bacterial action in the colon. Beyond its function in the intestinal digestion and fat absorption, this secondary bile acid has demonstrated other biological properties including vitamin D receptor modulation [42,43], proteasome regulation [44] and anti-proliferative and pro-apoptotic effects on cancer cells in vitro and in vivo [45,46,47]. 

It is important to note that the small molecule inhibitors of bacterial response regulators could have other targets in the cell, triggering bacterial death or virulence decrease by inhibiting and/or affecting the activities of several biomolecules at the same time. Thus, apigenin, one of the HsrA inhibitors and potent anti-*H*. *pylori* bactericidal flavonoid [16], also functions as an inhibitor of the enzymes D-alanine:D-alanine ligase [48] and β-hydroxyacyl-acyl carrier protein dehydratase [49]. Similarly, the lipophilic flavonoid hesperetin inhibited HsrA activity [16], but also affected the integrity of *H*. *pylori* cells [50]. Here, we demonstrated that LCA acts as a U-binder of the essential response regulator ArsR and completely inhibits its DNA binding activity according to EMSA experiments. Despite the fact that inhibition of the ArsR physiological function could be enough to interfere with *H. pylori* cell viability, several studies indicate that certain steroids could impair the viability of this bacteria via membrane injury, due to the inhibition of cholesterol assimilation [51]. However, MIC values of most bile acids against *H. pylori* appeared in the range of 200 to 400 mg/L, including CDCA, the precursor of LCA [52]. Hence, the putative membrane injury alone cannot account for the bactericidal effects of LCA observed in our experiments.

On the other hand, LCA showed partial synergisms with other anti-*H. pylori* drugs such as clarithromycin, levofloxacin and chrysin. Notably, this bile acid increased the bactericidal potency of clarithromycin up to 32 times against the *H. pylori* clarithromycin-resistant strain ATCC 700684. Previous studies have demonstrated synergistic action of LCA and its derivatives on the activities of other conventional antibiotics such as aminoglycosides [53].

## 5. Conclusions

A major challenge in the current antibiotic resistance crisis is the identification of novel microbial targets, essential for in vivo growth or pathogenicity, whose inhibitors can overcome the currently circulating resistome of human pathogens. In previous works, we validated a new effective anti-*H. pylori* therapeutic target, the essential response regulator HsrA [16,17]. The results of the present study strongly support the use of a novel therapeutic target against *H. pylori*, the essential response regulator ArsR. Non-antibiotic low-molecular weight compounds from a repurposing chemical library bound to ArsR and noticeably inhibited its essential biological activity. Some of these compounds, such as the human secondary bile acid LCA, exhibit bactericidal activity against antibiotic-resistant strains of *H. pylori* and partially synergizes with other conventional antibiotics, including clarithromycin and levofloxacin. The results of our experiments enhance the battery of novel therapeutic tools to include in personalized combinatory strategies against refractory infections caused by multidrug-resistant *H. pylori* strains.

## Figures and Tables

**Table 1:** ArsR binders identified by HTS of the Prestwick Chemical Library^®^ using a fluorescence-based thermal shift assay.

Compound	Δ*T*_m_ (°C) *^a^*	Therapeutic Group *^b^*	Chemical Structure
Lorglumide sodium salt	−1.6	cholecystokinin antagonist	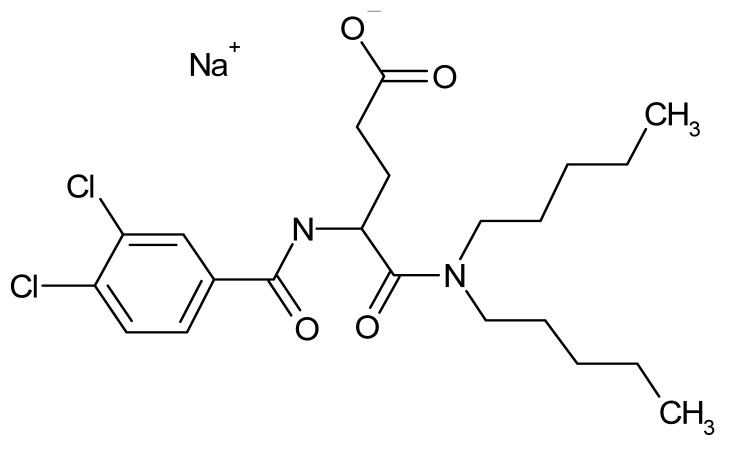
Meclofenamic acid sodium salt monohydrate	−1.7	anti-inflammatory, antipyretic	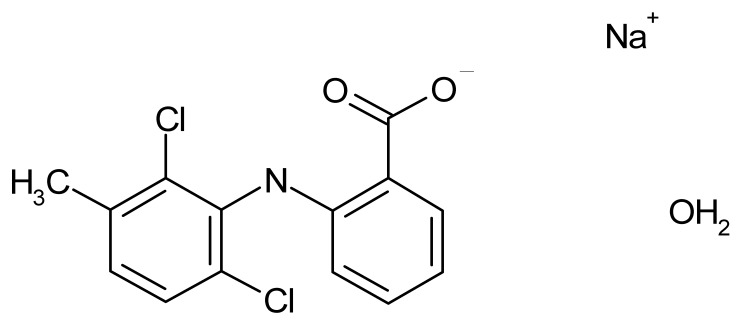
Miconazole	−2.0	antifungal	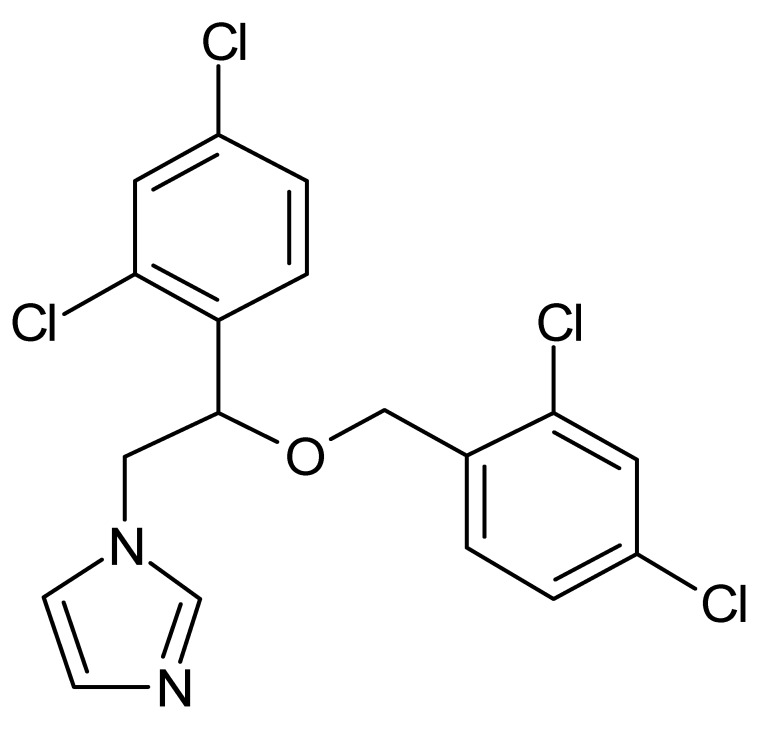
Tiratricol	−2.3	hypocholesterolemic, antityroidic hormone	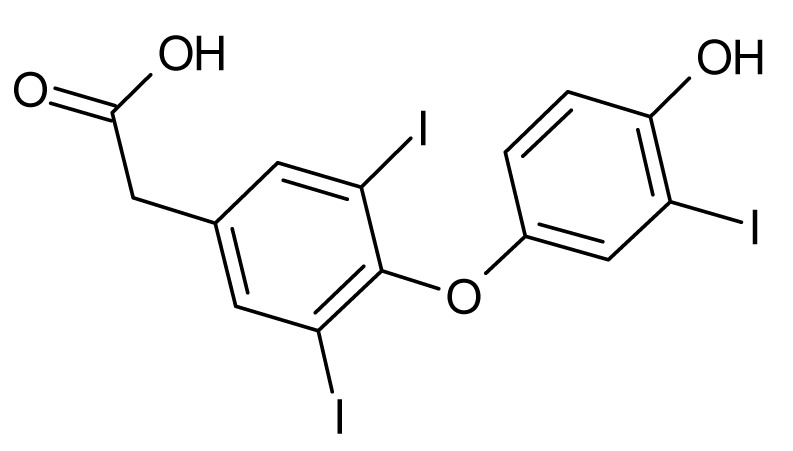
Propidium iodide	−2.7	antibacterial	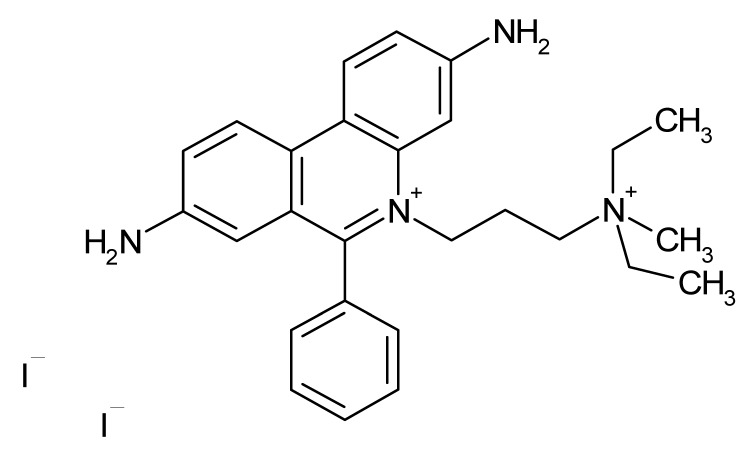
Diethylstilbestrol	−2.9	estrogen, antineoplastic	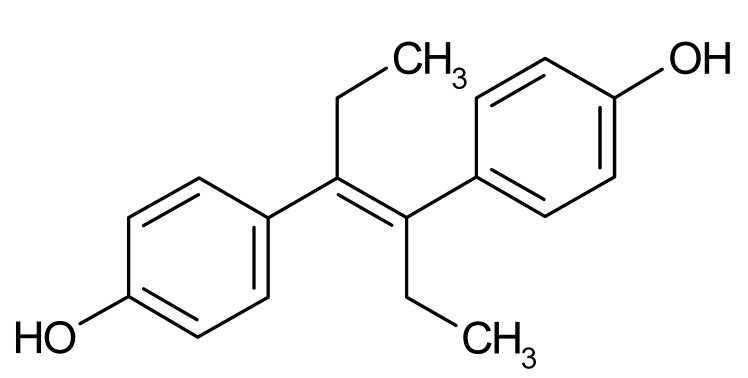
Dequalinium dichloride	−3.0	antibacterial	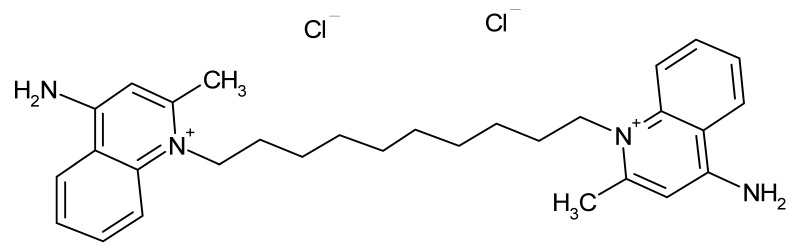
Lithocholic acid	−3.1	cholagogue, choleretic	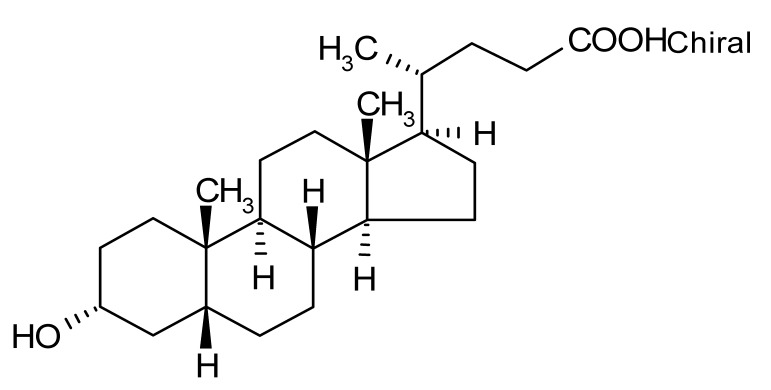
Simvastatin	−3.5	antihyperlipidemic	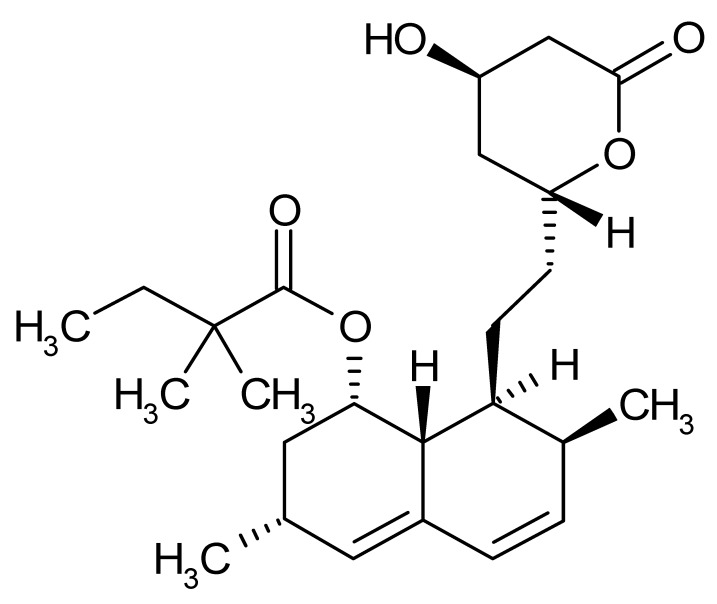
Dienestrol	−6.8	non-steroidal estrogen	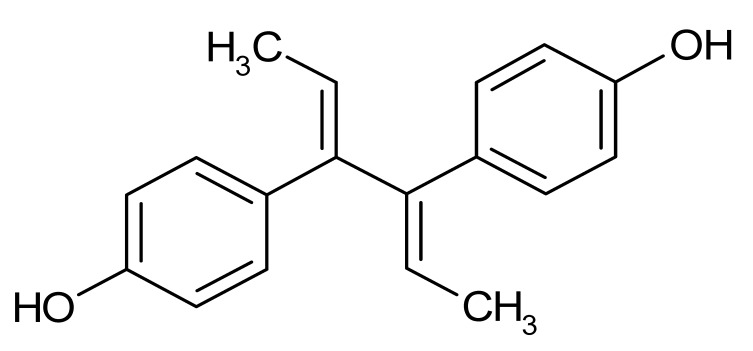

*^a^*All ArsR binders trigger a decrease in the *T*_m_ value of protein-ligand complex with respect to the mean *T*_m_ value of controls (protein + DMSO), thereby acting as destabilizers or U-binders. In all cases, Δ*T*_m_ > two-fold standard deviation of controls. *^b^* According to the Prestwick Chemical Library® database (http://www.prestwickchemical.com).

**Table 2:** Thermodynamic analysis of the interaction between ArsR and its inhibitors by ITC.

ArsR Inhibitor	ITC *^a^*			
	*n*	*K*_d_(µM)	ΔH(kcal/mol)	ΔG(kcal/mol)
Tiratricol	0.74	8.6	−14.4	−6.9
Propidium iodide	0.92	18	−19.1	−6.5
Lithocholic acid	0.69	4.9	−7.8	−7.2
Lorglumide sodium salt	0.78	0.67	1.8	−8.4

*^a^*Absolute error in n is 0.06, relative error in *K*_d_ is 40%, absolute error in ΔH is 0.4 kcal/mol, absolute error in ΔG is 0.2 kcal/mol.

**Table 3:** Minimal inhibitory and bactericidal concentrations of ArsR inhibitors against different strains of *H. pylori.*

Drug	MIC (MBC), mg/L		
	ATCC 700392	ATCC 43504 (MTZ-R)	ATCC 700684 (CLR-R)
Tiratricol	64 (64)	64 (64)	64 (64)
Propidium iodide	16 (32)	16 (32)	16 (32)
Lithocholic acid	32 (32)	32 (32)	32 (32)
Lorglumide	64 (64)	64 (64)	64 (128)
Metronidazole	1 (2)	64 (128)	1 (2)
Clarithromycin	≤ 0.12 (≤ 0.12)	≤ 0.12 (≤ 0.12)	64 (128)

MTZ-R, metronidazole resistant strain. CLR-R, clarithromycin resistant strain.

**Table 4:** Combinatory effect of lithocholic acid (LCA) with several drugs against *H. pylori.*

Strain	Combination Tested	FIC_drug_	FIC_LCA_	FICI *^a^*	Interaction *^b^*
ATCC 700684	LCA + Clarithromycin	0.031	0.5	0.53	Additive
ATCC 43504	LCA + Metronidazole	1	1	2	No interaction
ATCC 700392	LCA + Levofloxacin	0.5	0.5	1	Additive
ATCC 700392	LCA + Chrysin	0.25	0.5	0.75	Additive

*^a^*Fractional inhibitory concentration index (FICI) could be calculated as: FIC_A_ (MIC_A in the presence of B_/MIC_A alone_) + FIC_B_ (MIC_B in the presence of A_/MIC_B alone_). *^b^*According to the FICI value, the interaction between two compounds against a particular bacterial strain can be classified as: synergy (FICI ≤ 0.5), additive (FICI >0.5 to ≤1), no interaction or neutral (FICI > 1 to ≤ 4), and antagonism (FICI > 4).

## Supplementary Materials

The following are available online at https://www.mdpi.com/2076-2607/8/4/503/s1, Figure S1: Isothermal titration calorimetry experiments for the interaction of the *H. pylori* ArsR response regulator with its inhibitors. In the figure, upper panels show the ITC thermograms while lower panels show the binding isotherms.
