# Supplementary material for: Small Molecule Inhibitors of the Response Regulator ArsR Exhibit Bactericidal Activity against Helicobacter pylori
Source: Microorganisms. 2020 Apr 1;8(4):503. doi: 10.3390/microorganisms8040503 (PMC7232201; doi:10.3390/microorganisms8040503)
Supplement: Supplementary file 1 [file microorganisms-08-00503-s001.pdf]

# Supplementary Materials: Small molecule inhibitors of the response regulator ArsR exhibit bactericidal activity against *Helicobacter pylori*

**Andrés González, Javier Casado, Eduardo Chueca, Sandra Salillas, Adrián Velázquez-Campoy, Javier Sancho and Ángel Lanas.**

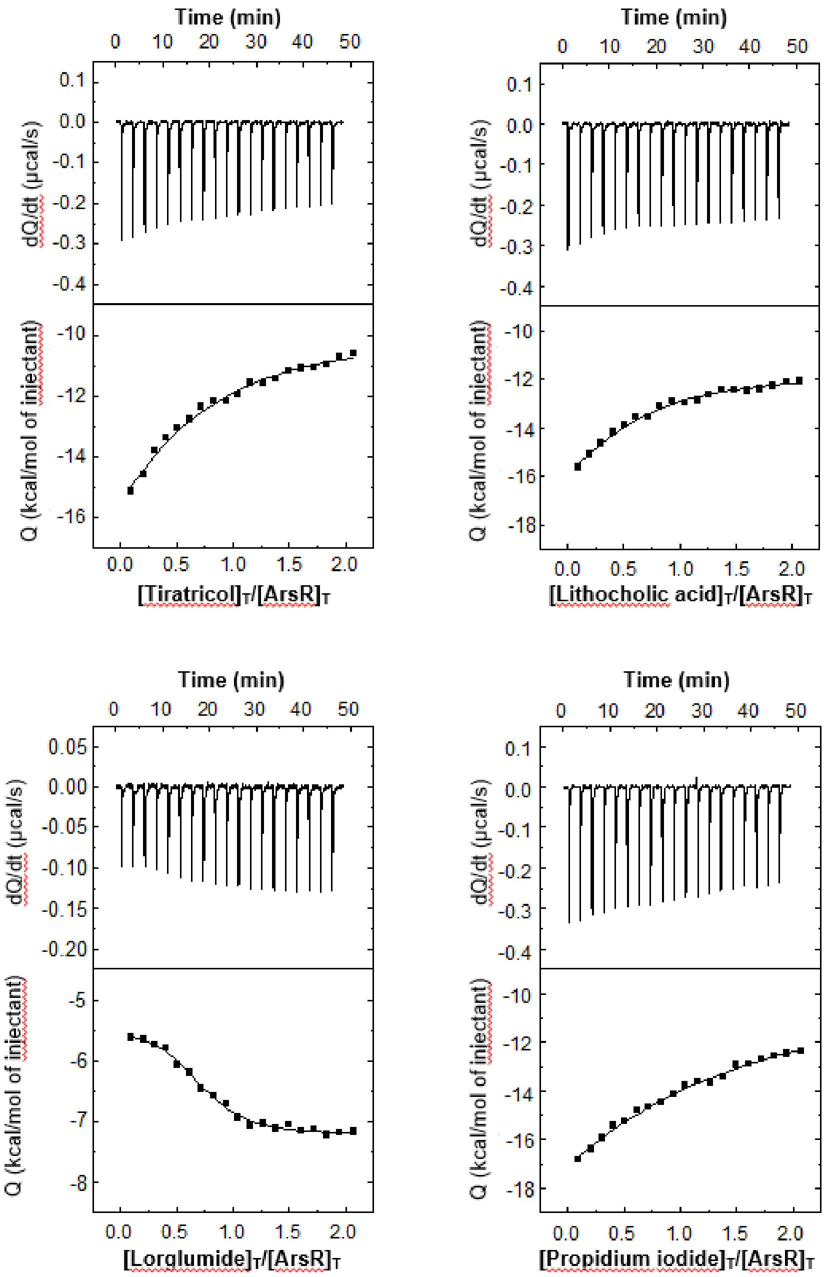


**Figure S1.** Isothermal titration calorimetry experiments for the interaction of the *H. pylori* ArsR response regulator with its inhibitors. In the figure, upper panels show the ITC thermograms while lower panels show the binding isotherms.
